# Green Synthesis of Silver Nanoparticles from *Melissa officinalis* Flower: Evaluation of Antimicrobial, Antioxidant, and Photocatalytic Activity

**DOI:** 10.3390/molecules31183300

**Published:** 2026-09-17

**Authors:** Emre Erden Kopar

**Affiliations:** Biochemistry Department, Faculty of Science, Ege University, İzmir 35100, Türkiye; emre.erden@ege.edu.tr; Tel.: +90-(232)-311-2455

**Keywords:** *Melissa officinalis* flower, green synthesis, AgNPs, antioxidant, antimicrobial, photocatalytic

## Abstract

Silver nanoparticles (AgNPs) synthesized via green approaches have attracted considerable attention because of their eco-friendly production and versatile biological and photocatalytic properties. In this study, *Melissa officinalis* flower extract was employed as a natural reducing, capping, and stabilizing agent for the green synthesis of AgNPs. HPLC-DAD analysis revealed that the flower extract exhibited a diverse phytochemical profile, including phenolic acids and flavonoids, which may contribute to the reduction of Ag^+^ ions and the stabilization of the synthesized AgNPs. The synthesized nanoparticles were characterized using UV–Vis spectroscopy, ATR-FTIR, SEM, DLS, zeta potential analysis, and XRD. The AgNPs exhibited predominantly spherical to hemispherical morphology with an average particle size of 40 nm and a characteristic surface plasmon resonance peak at 414 nm. DLS analysis revealed a particle size of 111.2 nm and a Z-average hydrodynamic diameter of 296.0 nm, while the zeta potential of −73.2 mV suggested high colloidal stability. XRD analysis confirmed the formation of a face-centered cubic (fcc) crystalline structure. The synthesized AgNPs exhibited photocatalytic activity, achieving degradation efficiencies of 93.6 ± 0.3% for methylene blue and 44.8 ± 0.6% for Coomassie Brilliant Blue R-250 after 180 min. In addition, the nanoparticles exhibited considerable antioxidant activity in the DPPH assay and antimicrobial activity against *Escherichia coli*, *Klebsiella pneumoniae*, *Staphylococcus aureus*, and *Candida albicans*. Overall, the findings indicate that AgNPs synthesized from *M. officinalis* flowers via the green synthesis method exhibited photocatalytic, antioxidant, and antimicrobial activities under the experimental conditions studied. These findings suggest that the synthesized AgNPs could be considered a potential material, particularly for dye removal and antimicrobial applications.

## 1. Introduction

Nanotechnology is a rapidly evolving field of research in areas such as biology, chemistry, and materials science, driven by the growing demand for materials and devices on the nanometer scale [1]. Nanoparticles—generally defined as materials in the 1–100 nm size range—are attracting significant interest due to their unique physicochemical properties and diverse applications [2]. Among metal nanoparticles, silver nanoparticles (AgNPs) are widely studied due to their high surface area-to-volume ratio, characteristic surface plasmon resonance (SPR), chemical stability, and ease of surface functionalization [3]. In addition to these properties, their broad-spectrum antimicrobial and catalytic activities have enabled the use of AgNPs in biomedical, environmental, and catalytic applications [4,5]. Although AgNPs can be synthesized using various physicochemical methods, these methods may involve the use of hazardous reagents and have adverse effects on the environment and living organisms [6]. For this reason, green synthesis—which relies on naturally abundant and renewable resources—is attracting increasing attention as an alternative approach [7].

The properties and stability of nanoparticles obtained through biosynthesis may vary depending on the biological material used and the conditions applied. The synthesis of nanoparticles derived from biological sources (plants, microorganisms, fungi, algae, etc.) is considered safer compared to traditional/synthetic approaches [8]. These approaches can facilitate the production of well-dispersed and morphologically controllable nanoparticles while offering scalable and cost-effective processes [8,9]. However, the use of biological sources does not mean that all stages of the synthesis process are completely free of organic solvents or other chemical reagents.

Plants have been the most preferred materials in green synthesis applications. The ability of plant extracts to alter the oxidation state of metals has opened up new possibilities for applications such as the synthesis of metallic nanoparticles. These plant extracts have high potential for the attraction of metal ions and their accumulation without harming normal metabolic activities in cells [10]. Plant-based sources are considered advantageous in nanoparticle synthesis due to their accessibility, low cost, environmental compatibility, and the presence of bioactive phytochemicals that can facilitate nanoparticle formation in various sizes and shapes. These properties make plant materials ideal candidates for sustainable nanoparticle production platforms [9,11]. Phenolic acids, flavonoids, terpenoids, alkaloids, and other secondary metabolites found in plant extracts act as natural capping and stabilizing agents on the surface of synthesized nanoparticles, in addition to reducing metal ions [12]. This can contribute to the formation of nanoparticles with controlled size, shape, and high colloidal stability [12].

*Melissa officinalis* L. (lemon balm), a perennial aromatic and medicinal plant belonging to the Lamiaceae (Labiatae) family, grows naturally in Europe, Western Asia, and the Mediterranean basin [13]. It has been used for many years in traditional medicine for its sedative, anxiolytic, antispasmodic, antiviral, antibacterial, and anti-inflammatory properties [13,14,15]. The plant’s biological activities are largely attributed to rosmarinic acid, caffeic acid, chlorogenic acid, gallic acid, ferulic acid, and phenolic compounds such as luteolin, apigenin, and quercetin. These phytochemical compounds not only exhibit strong antioxidant properties but also function as natural reducing and coating agents that facilitate the reduction of Ag^+^ ions and play a role in stabilizing the synthesized nanoparticles [16].

*M. officinalis* flowers, in particular, are considered one of the suitable biological sources for nanoparticle synthesis because they are rich in phenolic compounds and natural antioxidants [17]. This plant is rich in phenolic compounds and antioxidants that contribute to protective effects against cellular damage caused by oxidative stress. The antioxidant activity of *M. officinalis* flowers plays an important role in supporting cellular renewal and reducing the risk of chronic diseases, including cardiovascular disorders and various types of cancer. Thanks to these bioactive compounds, *M. officinalis* flowers are a promising natural resource for biomedical and nanotechnological applications [18].

Studies in the literature on the synthesis of green AgNPs using *M. officinalis* demonstrate that this plant is an effective source of biological reduction and stabilization, thanks to its phenolic and flavonoid compounds. Ruíz-Baltazar et al. [19] and Fierascu et al. [20] reported that AgNPs synthesized using *M. officinalis* extract exhibited significant antimicrobial activity and that plant-derived phytochemicals played a key role in the formation and stabilization of the nanoparticles. In subsequent studies, Nayeri et al. [21] demonstrated that the extraction method plays a decisive role in the physicochemical properties and biological activity of the nanoparticles, whilst Motafeghi et al. [22] showed that nanomaterials derived from *M. officinalis* offer promising results in terms of anticancer, antioxidant and biosafety properties. Coskun and Kapdan [23] highlighted the nanobiotechnological potential of this plant by reporting that AgNPs increased the production of secondary metabolites in *M. officinalis* cultures. However, whilst the majority of existing studies have focused on the biological properties of nanoparticles synthesized using leaf extracts, research on the biological and, in particular, environmental applications of AgNPs synthesized exclusively from *M. officinalis* flowers remains limited.

Although there are numerous studies in the literature on AgNPs synthesized using various plant extracts, there are very few studies that optimize the synthesis conditions for AgNPs synthesized using *M. officinalis* flower extract, perform a detailed physicochemical characterization, and evaluate their antimicrobial, antioxidant, and photocatalytic properties together within the same study. The main innovation of this study is the integrated approach to the synthesis optimization and physicochemical characterization of AgNPs synthesized exclusively from *M. officinalis* flowers, combined with antimicrobial, antioxidant, and photocatalytic evaluations. In particular, the photocatalytic degradation of methylene blue (MB) and Coomassie Brilliant Blue R-250 (CBB R-250) in conjunction with the biological activities of AgNPs synthesized from *M. officinalis* flowers remains insufficiently explored. Therefore, the present study was designed to address this gap by optimizing the synthesis conditions and characterizing AgNPs synthesized exclusively from *M. officinalis* flowers and evaluating their antimicrobial, antioxidant, and photocatalytic activities toward MB and CBB R-250.

This study focuses on the green synthesis of AgNPs using an extract of the *M. officinalis* flower plant. The nanoparticles synthesized using the green synthesis method were characterized using ultraviolet-visible spectroscopy (UV-Vis), Fourier transform infrared spectroscopy (ATR-FTIR), scanning electron microscopy (SEM), and X-ray diffraction (XRD). Additionally, the antimicrobial properties of AgNPs were investigated using Gram-negative (*Escherichia coli*, *Klebsiella pneumoniae*) and Gram-positive (*Staphylococcus aureus*) bacteria and fungi (*Candida albicans*). In addition, the antioxidant activities of the synthesized nanoparticles were evaluated using the DPPH radical scavenging method, and their photocatalytic degradation activity for MB and CBB R-250 dyes was also investigated. Therefore, the aim of this study was to optimize the green synthesis conditions for AgNPs using *M. officinalis* flower extract, characterize the synthesized nanoparticles, and evaluate their antimicrobial, antioxidant, and photocatalytic activities against MB and CBB R-250 dyes.

## 2. Results and Discussion

### 2.1. Evaluation of the Phytochemical Composition of M. Officinalis Flower Extract

**Figure 1:**
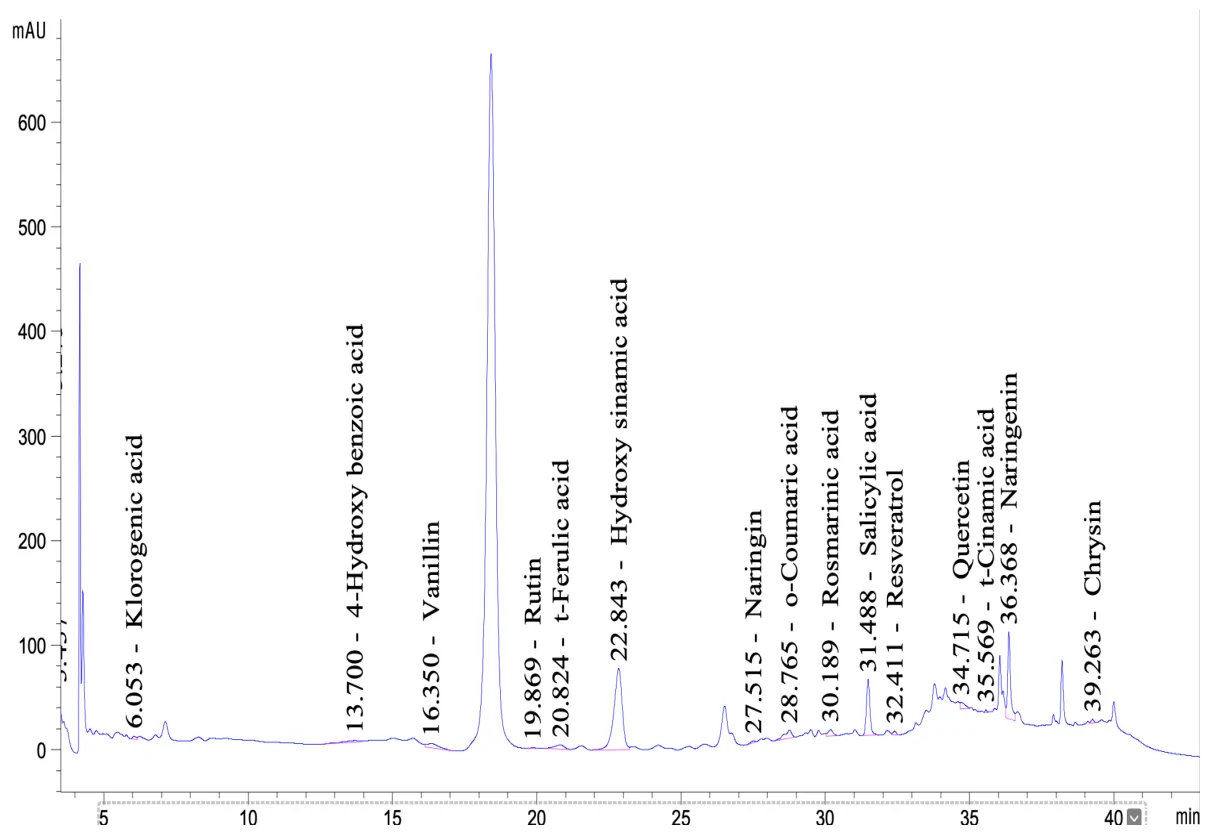
HPLC-DAD profile and predicted compounds of the *M. officinalis* flower extract.

HPLC-DAD analysis of an 80% methanol extract of *M. officinalis* flowers revealed a phytochemical profile rich in phenolic acids and flavonoids (Figure 1). A total of 15 compounds were identified in the chromatogram. Among the identified compounds, hydroxycinnamic acid (31.916 ng/µL), naringenin (23.750 ng/µL), and salicylic acid (21.832 ng/µL) were present at the highest levels, followed by chrysin (7.621 ng/µL), vanillin (4.370 ng/µL), o-coumaric acid (3.075 ng/µL), trans-ferulic acid (2.814 ng/µL), quercetin (2.289 ng/µL), rosmarinic acid (2.229 ng/µL), and 4-hydroxybenzoic acid (2.111 ng/µL). Chlorogenic acid (1.347 ng/µL), naringin (1.269 ng/µL), resveratrol (0.849 ng/µL), trans-cinnamic acid (0.206 ng/µL), and rutin (0.161 ng/µL) were detected at comparatively lower levels. The profile obtained was generally consistent with previous reports describing flavonoids such as rutin, quercetin, naringin, and naringenin, as well as phenolic acids including rosmarinic, chlorogenic, caffeic, and ferulic acids in the phenolic composition of *M. officinalis* flowers [14,22,24,25]. In particular, various HPLC studies have consistently shown that rosmarinic acid is a common and important phenolic compound in *M. officinalis* samples [26,27,28]. However, the fact that rosmarinic acid was not the predominant compound in the present study may be related to the different extraction conditions used—specifically, the use of only flowers, unlike the leaf/aerial parts and infusion samples reported in the literature [27,29]. This quantitative pattern also differs from other plant-part-specific studies. Fierascu et al. reported rutin as the dominant flavonoid (1462.997 mg/kg) in ethanolic extracts of *M. officinalis* leaves, whereas in the present flower-derived extract rutin was detected only at trace levels and quercetin remained comparatively low (2.289 ng/µL) [20]. This discrepancy further supports the view that flavonoid and phenolic acid distribution in *M. officinalis* is strongly plant-part- and solvent-dependent rather than reflecting a fixed chemotype.

### 2.2. Synthesis of AgNPs

**Figure 2:**
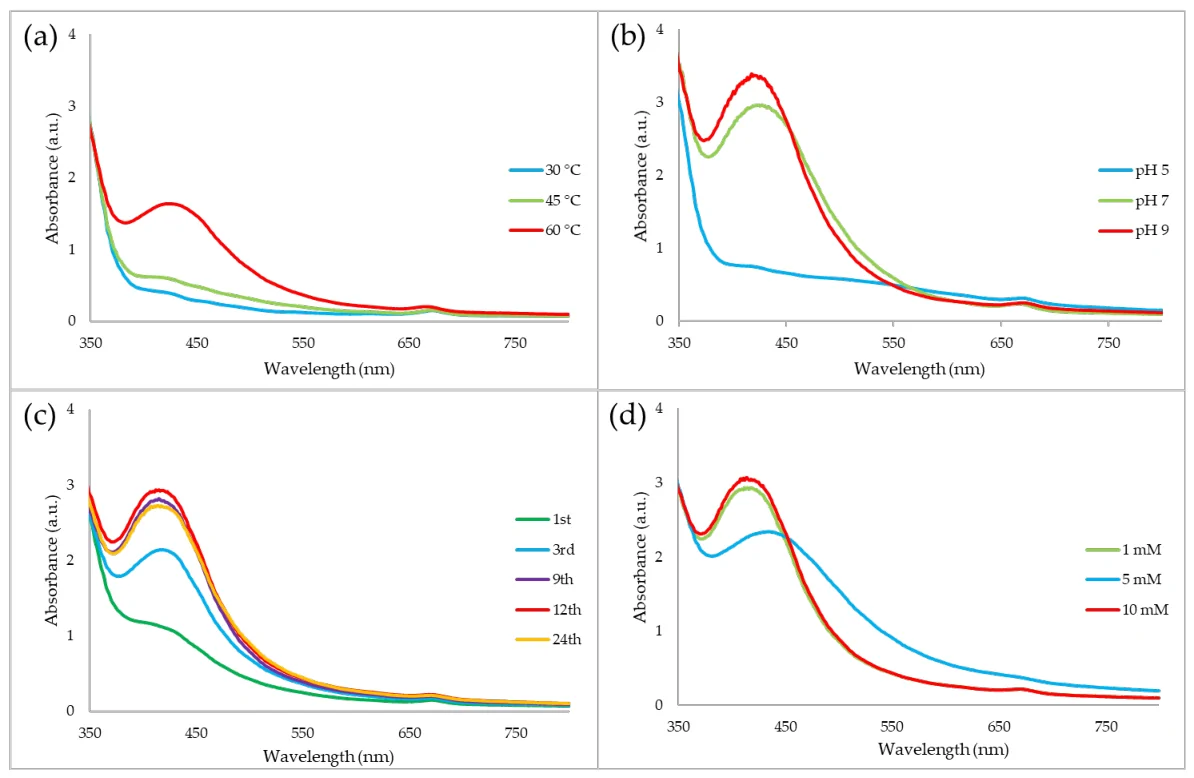
Optimization of AgNP synthesis conditions using *M. officinalis* flower extract. (**a**) Effect of temperature, (**b**) effect of initial pH, (**c**) effect of reaction time, and (**d**) effect of AgNO_3_ concentration on the UV–Vis spectra of the synthesized AgNPs.

The optimization studies showed that UV-Vis spectral characteristics of AgNPs varied depending on temperature, pH, reaction time, and precursor concentration (Figure 2). Among the tested conditions, the highest SPR absorbance was observed at 60 °C, and this temperature was therefore selected as the optimum condition (Figure 2a). Similarly, the highest SPR absorbance was observed at pH 9, which was therefore selected as the optimum pH (Figure 2b). In terms of reaction time, the SPR band intensity increased up to 12 h, while no substantial further increase was observed at 24 h. Therefore, 12 h was selected as the optimum reaction time (Figure 2c). Additionally, the highest SPR absorbance was obtained at 10 mM AgNO_3_, which was consequently selected as the optimum precursor concentration (Figure 2d). Based on the results of the UV–Vis analysis, the optimal synthesis conditions were determined to be 60 °C, pH 9, 12 h, and 10 mM AgNO_3_.

### 2.3. Characterization of AgNPs

**Figure 3:**
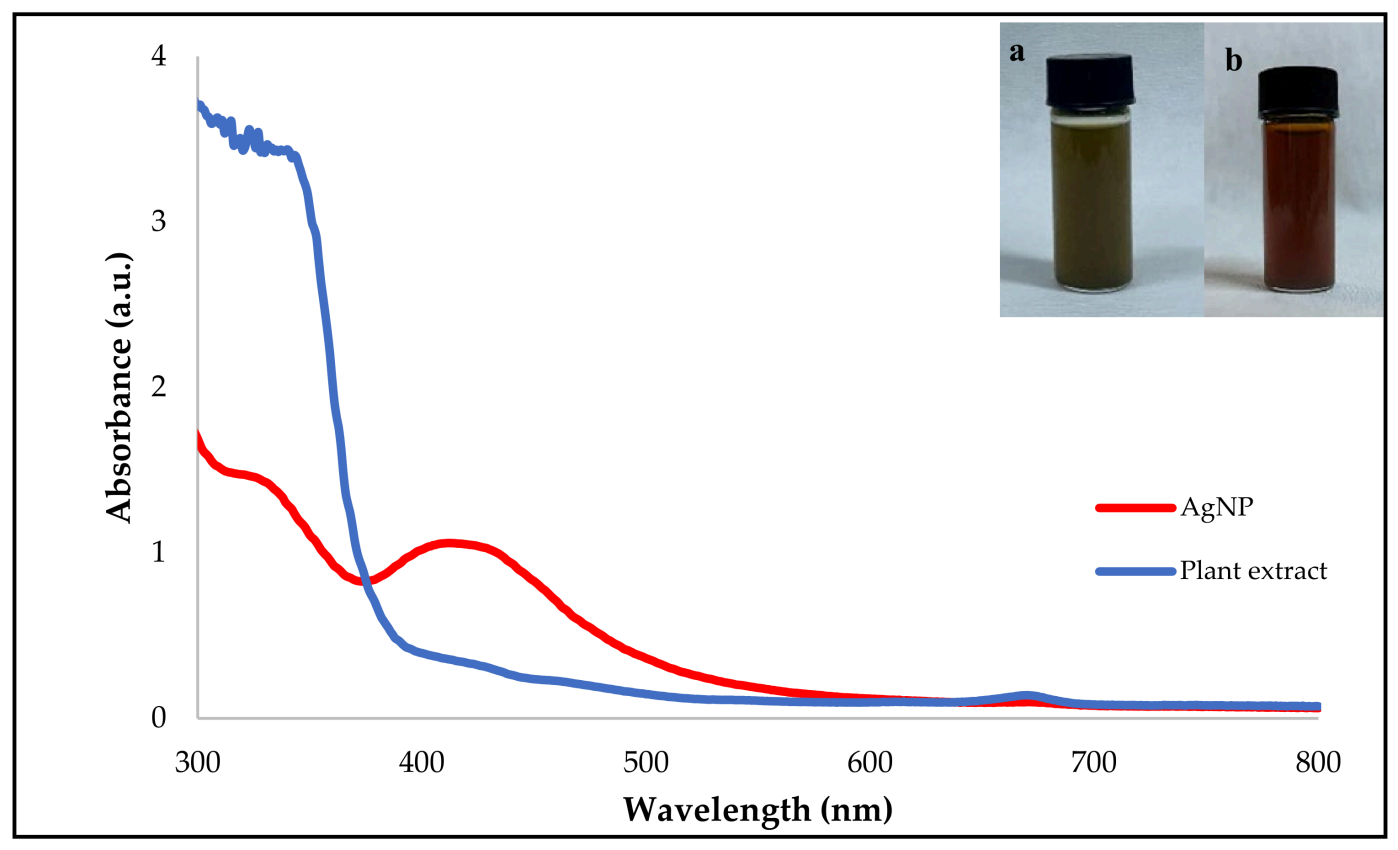
UV-Vis spectrum (300–800 nm) scan of AgNPs synthesized from *M. officinalis* flowers using the green synthesis method (color change over time in the AgNPs synthesis process using the green synthesis method from *M. officinalis* flowers [(**a**) start of synthesis, (**b**) end of synthesis]).

UV-visible spectrophotometry is a reliable analytical technique widely used for the characterization of metallic nanoparticles. Twelve hours after synthesis, a distinct color change from light yellow to dark brown was observed in the reaction mixture, indicating the reduction of Ag^+^ ions to metallic silver (Ag^0^) and the formation of AgNPs. This visual observation was further confirmed by UV–Vis analysis, in which the synthesized AgNPs exhibited a characteristic SPR absorption peak at 414 nm (Figure 3) [30]. In contrast, the UV–Vis spectrum of the *M. officinalis* flower extract showed strong absorption only in the UV region (300–360 nm), which can be attributed to naturally occurring phenolic compounds, flavonoids, and other phytochemicals, while no characteristic SPR band was detected in the visible region. The appearance of a distinct absorption peak at 414 nm after the reaction therefore confirms that the SPR band originated from the formation of AgNPs rather than from the phytochemical constituents of the plant extract. This finding is consistent with the typical absorption range reported for green-synthesized AgNPs (400–450 nm) [31]. The position and intensity of the SPR band are known to depend on several factors, including the size, shape, size distribution, degree of agglomeration of the nanoparticles, and the phytochemical composition of the plant extract employed during the synthesis process.

**Figure 4:**
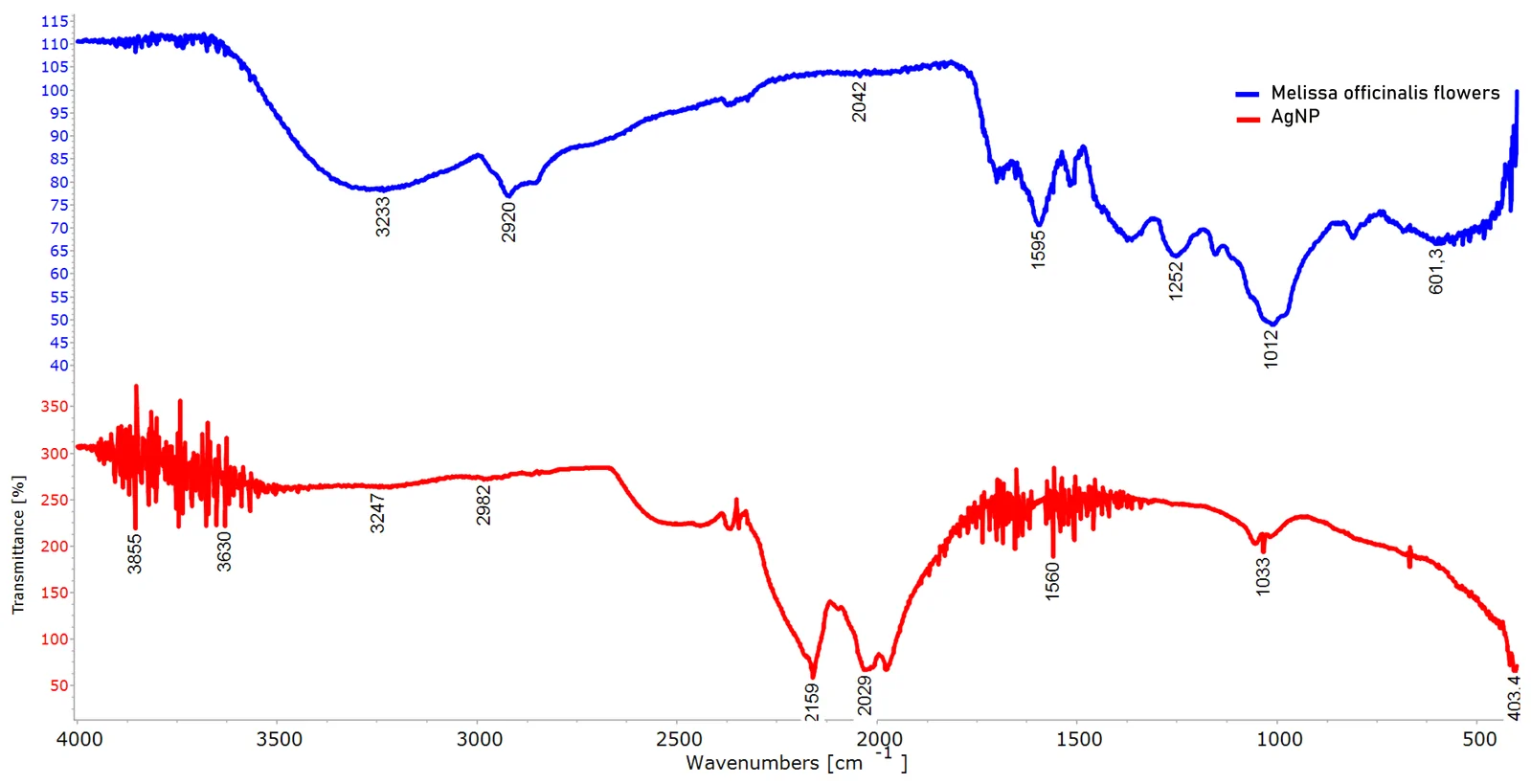
ATR-FTIR spectra of *M. officinalis* flower extract and synthesized AgNPs.

The ATR-FTIR spectra shown in Figure 4 reveal distinct differences at the functional group level between the *M. officinalis* flower extract and the AgNPs synthesized using this extract, thereby supporting the green synthesis mechanism.

While the broad band observed in the extract spectrum at 3233 cm^−1^ corresponds to the –OH stretching vibrations of phenolic compounds and flavonoids, the shift and intensity change in this band in the AgNP spectrum indicate that hydroxyl groups play a role in the reduction of Ag^+^ ions and their binding to the nanoparticle surface [32,33]; similarly, the preservation of aliphatic C–H vibrations at 2919 cm^−1^ suggests that terpenoids and lipid-like compounds participate in the post-synthesis stabilization process [34], the shift in C=O and/or aromatic C=C bands in the 1653 cm^−1^ in the AgNP spectrum indicates that proteins and phenolic structures have complexed with metal ions [35]. Furthermore, changes in the C–O and C–N vibrations observed in the 1032 cm^−1^ support the view that polysaccharides and other oxygen-containing functional groups act as a coating material by binding to the nanoparticle surface [36]. The bands that become prominent at lower wavenumbers (<600 cm^−1^) indicate metal-ligand (Ag–O) interactions and confirm the formation of AgNPs [32,34,35]. When all findings are evaluated together, it is understood that the biomolecules present in *M. officinalis* flower extract play a dual role in the reduction of Ag^+^ ions and the stabilization of the resulting Ag^0^ nanoparticles, and that the resulting nanostructures are biologically coated.

**Figure 5:**
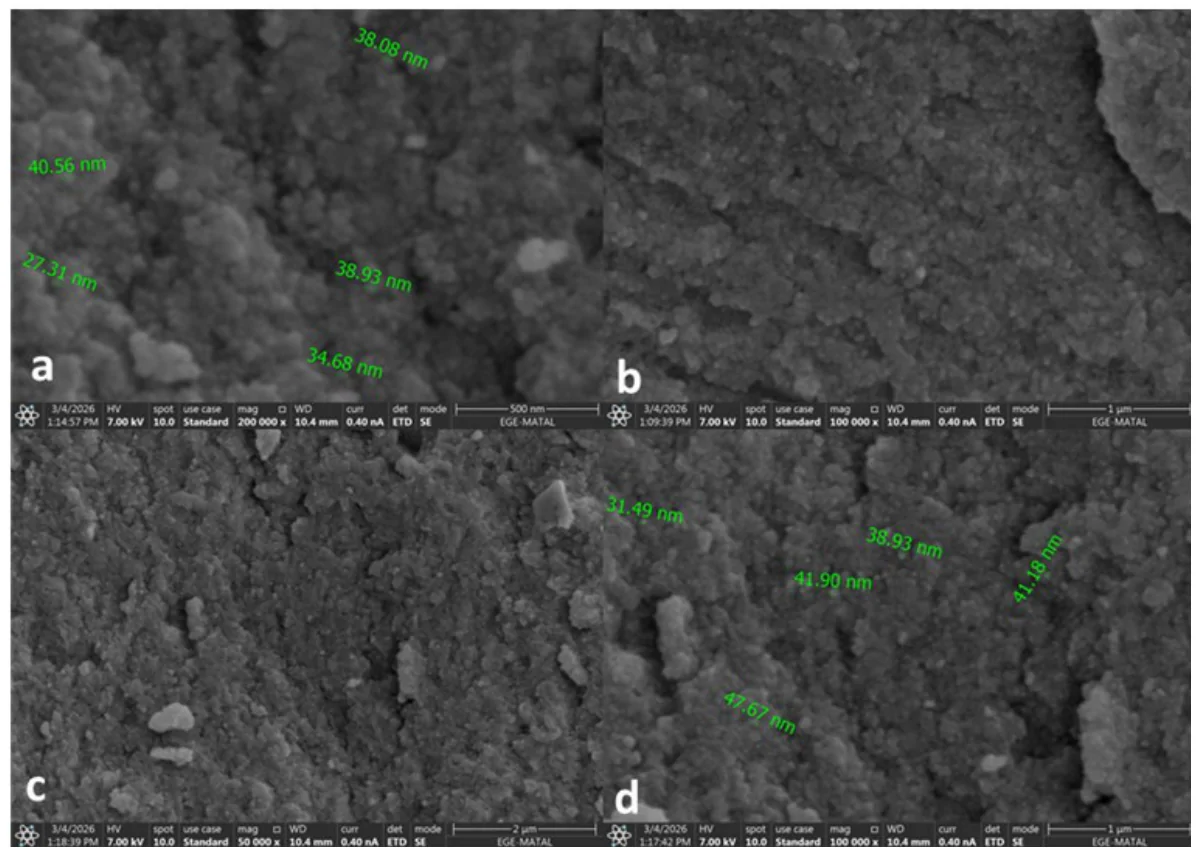
SEM images of AgNPs synthesized from *M. officinalis* flowers using the green synthesis method [(**a**) 500 nm, (**b**) 1 µm, (**c**) 2 µm, (**d**) 1 µm].

**Figure 6:**
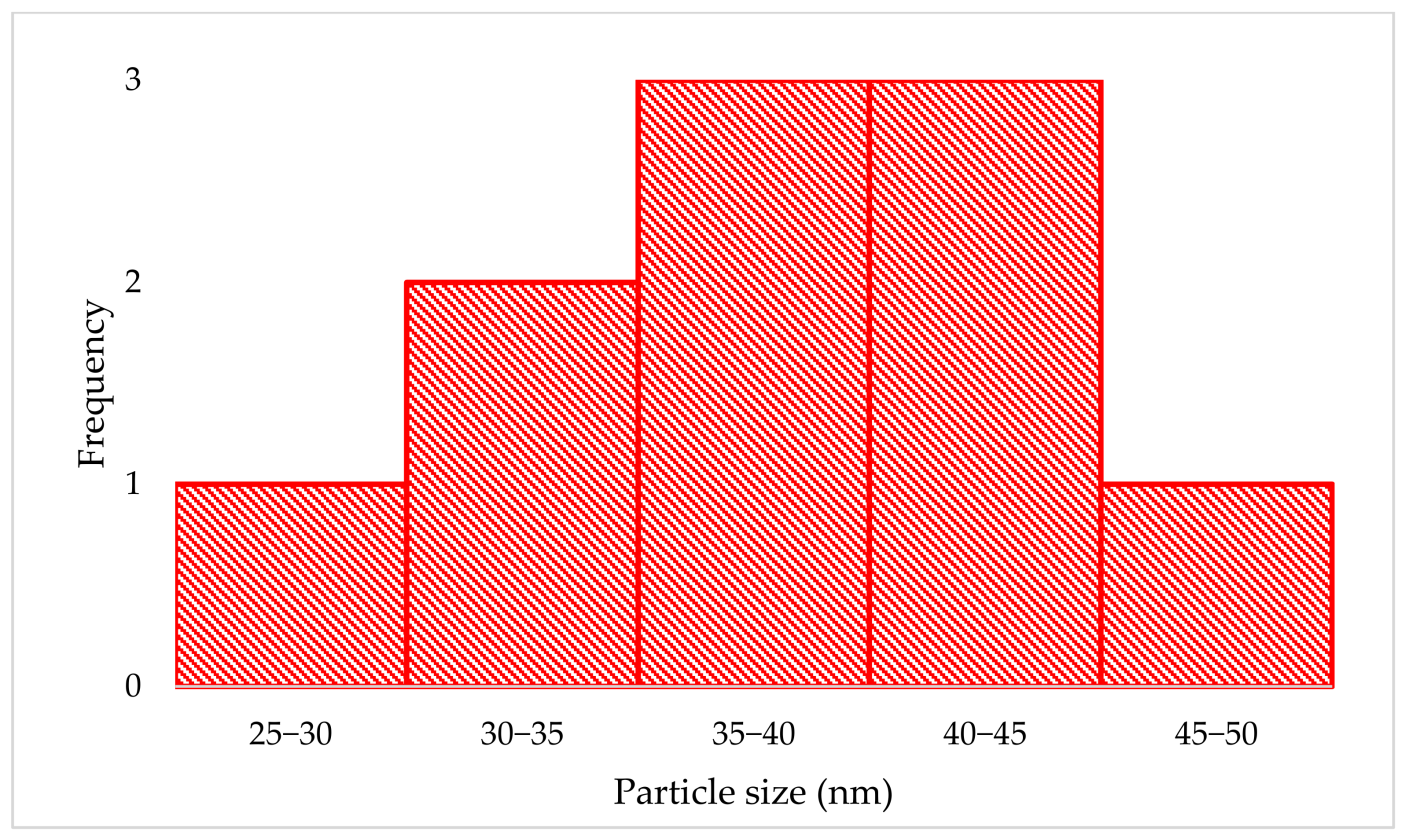
Particle-size distribution histogram of AgNPs synthesized using *M. officinalis* flower extract based on SEM measurements.

The morphological characteristics and external structural properties of AgNPs obtained by the green synthesis method with *M. officinalis* flower extract were evaluated by SEM analysis (Figure 5). SEM images reveal the size distribution, shape characteristics, and overall morphological structure of the synthesized NPs [37]. Particle size was determined by measuring individual nanoparticles—which were clearly distinguishable in representative SEM images—using the scale bar in the images as a reference. Results obtained at different magnifications revealed that the synthesized nanoparticles generally had a spherical or hemispherical morphology and showed a tendency to agglomerate. It was determined that the measured particle sizes ranged from 27.31 to 47.67 nm, which corresponds to a particle size range of approximately 27–48 nm. The corresponding particle-size distribution is presented as a histogram in Figure 6. 500 nm scale image shows that the particles are densely clustered and form aggregates consisting of nano-sized particles, while the 1 µm and 2 µm scale images show that the NPs exhibit a relatively homogeneous distribution across the surface but with a limited level of agglomeration commonly observed in biological synthesis processes. The limited level of aggregation observed in SEM images is a morphological feature commonly reported in the literature for metal nanoparticles produced by the green synthesis method [38]. This phenomenon stems from the tendency of the nanoparticles to interact with one another due to their high surface energy and van der Waals forces [39]. In addition, the phenolic compounds, flavonoids, and proteins found in *M. officinalis* flower extract coat the nanoparticle surface, providing natural stabilization, while the molecular interactions between these biomolecules may also contribute to particle aggregation to a certain extent [40]. However, the fact that the aggregation observed in the SEM images is not severe and that the primary nanoparticles retain their spherical morphology indicates that the synthesized AgNPs maintain their nanoscale structural integrity and are effectively stabilized by biological components [41]. The fact that aggregation remains at a limited level can be considered a significant advantage, as it helps to largely preserve the high specific surface area of nanoparticles, thereby enhancing their performance in antimicrobial and photocatalytic applications [42].

**Figure 7:**
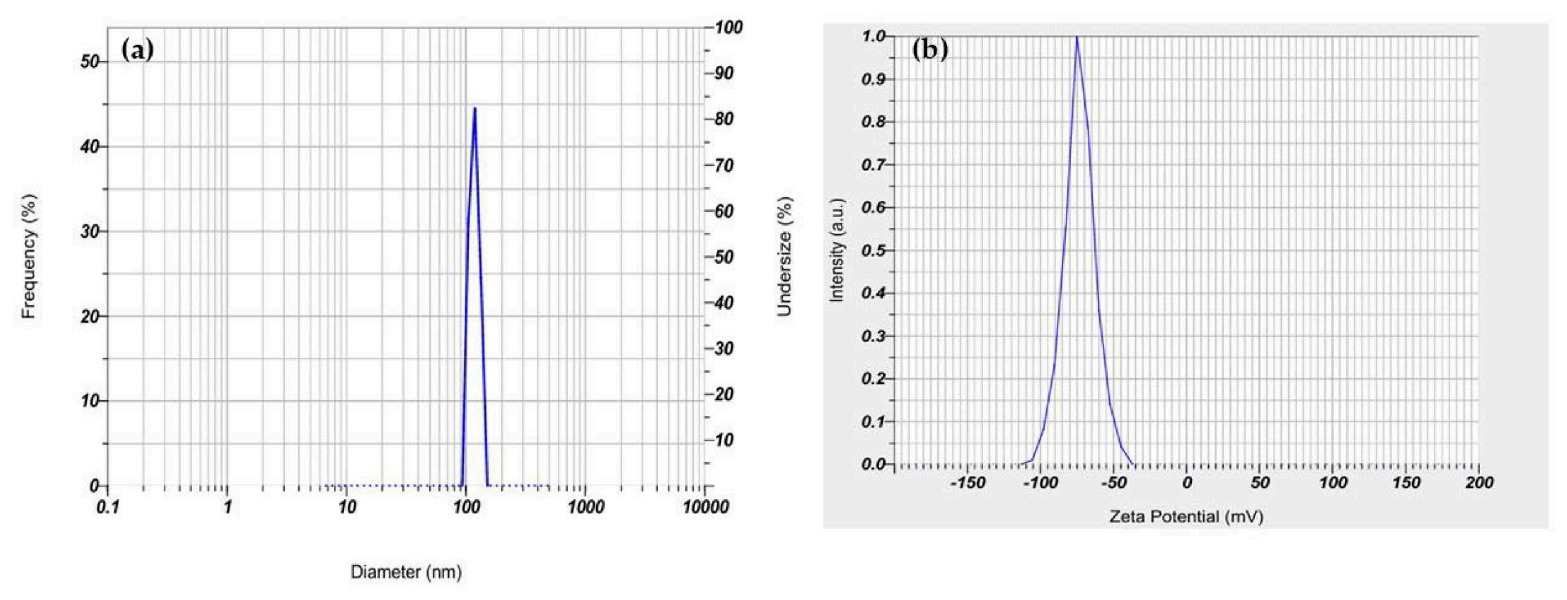
(**a**) DLS analysis showing the average particle size of AgNPs and (**b**) the zeta potential value indicating a surface charge of −73.2 mV.

DLS analysis showed that the AgNPs green-synthesized using *M. officinalis* extract had a dominant particle size of 111.2 nm, a Z-average (hydrodynamic diameter) of 296.0 nm, and a polydispersity index (PDI) of 0.523 (Figure 7a). Although a single dominant peak was observed, the relatively high PDI value (0.523) indicates a broad and heterogeneous particle-size distribution. However, the fact that the Z-average value is higher than the predominant particle size may be attributed to the DLS technique that measures the hydrodynamic diameter, which includes not only the metallic core but also the hydration layer surrounding the nanoparticle surface and the biomolecules adsorbed from the extract. In the zeta potential analysis, the dominant surface charge was determined to be −73.2 mV, indicating that the synthesized AgNPs possess high colloidal stability [43] (Figure 7b). It is believed that the high negative surface charge stems from the phenolic compounds, flavonoids, and other phytochemicals present in the *M. officinalis* extract binding to the nanoparticle surface and acting as natural coating and stabilizing agents [44]. These biomolecules may limit agglomeration by creating strong electrostatic repulsive forces between the nanoparticles and enhance the stability of the suspension [45]. When the DLS and zeta potential results are evaluated together, it can be concluded that AgNPs possess colloidal properties suitable for biological and photocatalytic applications [46].

**Figure 8:**
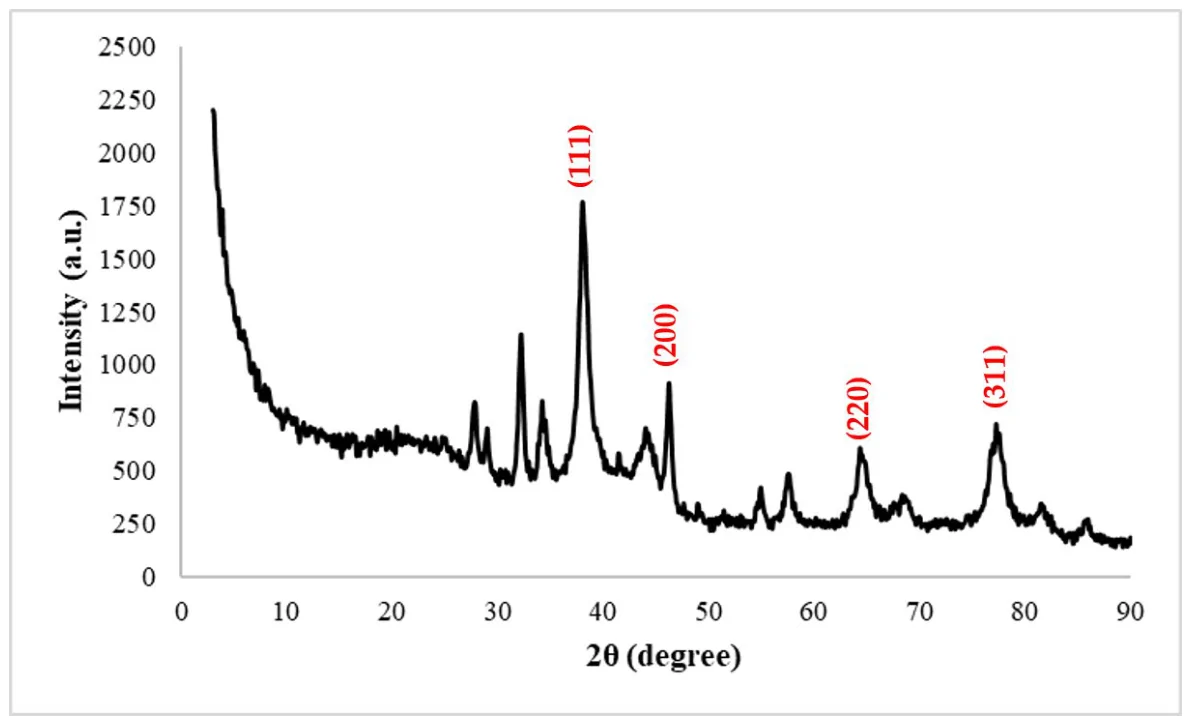
XRD pattern of AgNPs synthesized from *M. officinalis* flowers using the green synthesis method.

The XRD pattern of the AgNPs obtained via a green synthesis method using *M. officinalis* flower extract is characterized by distinct diffraction peaks which are observed at 2θ = 38.0°, 44.2°, 64.6°, and 77.5° (Figure 8). These peaks correspond to the (111), (200), (220), and (311) crystal planes, respectively, and were consistent with the characteristic reflections of metallic silver with a face-centered cubic (fcc) structure. The AgNP sample is consistent with the Joint Committee on Powder Diffraction Standards (JCPDS) 04-0783 standard [47]. A comparison of the experimental XRD pattern with the reference data from JCPDS No. 04-0783 confirmed that the observed diffraction peaks are consistent with the characteristic reflections of metallic silver with a fcc structure and showed that no distinct additional diffraction peaks attributable to crystalline impurity phases were observed [47]. The (111) plane at 2θ = 38.0°, which is the peak with the highest intensity, indicates the dominant crystal orientation frequently reported in the literature for AgNPs [48]. Additionally, the broad and low-intensity peaks were observed in the 2θ = 27.9–34.5° range. The background signal at low angles can be attributed to the presence of organic compounds derived from the plant extract and to the contribution of the amorphous phase. These results indicate that the synthesized nanoparticles contain a crystalline metallic silver phase and that the obtained XRD pattern is consistent with the data reported in the literature for AgNPs synthesized using plant extracts [47,49,50]. In addition, the average crystallite size of the synthesized AgNPs was calculated using the Scherrer equation based on the (111), (200), (220), and (311) planes, which exhibited the most prominent diffraction peaks. The crystallite size values corresponding to these diffraction peaks are presented in Table 1. The calculations revealed that the average crystallite size of the synthesized AgNPs was 9.07 ± 1.11 nm; this result confirms that the synthesized nanoparticles have a nanocrystalline structure. The fact that the average crystallite size determined by XRD analysis (9.07 ± 1.11 nm) is lower than the particle size observed in SEM analysis (27–48 nm) can be explained by the fact that the two techniques evaluate different size parameters; While XRD determines the size of the crystalline regions within the crystal structure that undergo coherent diffraction, SEM reveals the morphologically observed visible size of the particles.

### 2.4. Determination of Antioxidant Activity with DPPH Radical Scavenging Assay

**Figure 9:**
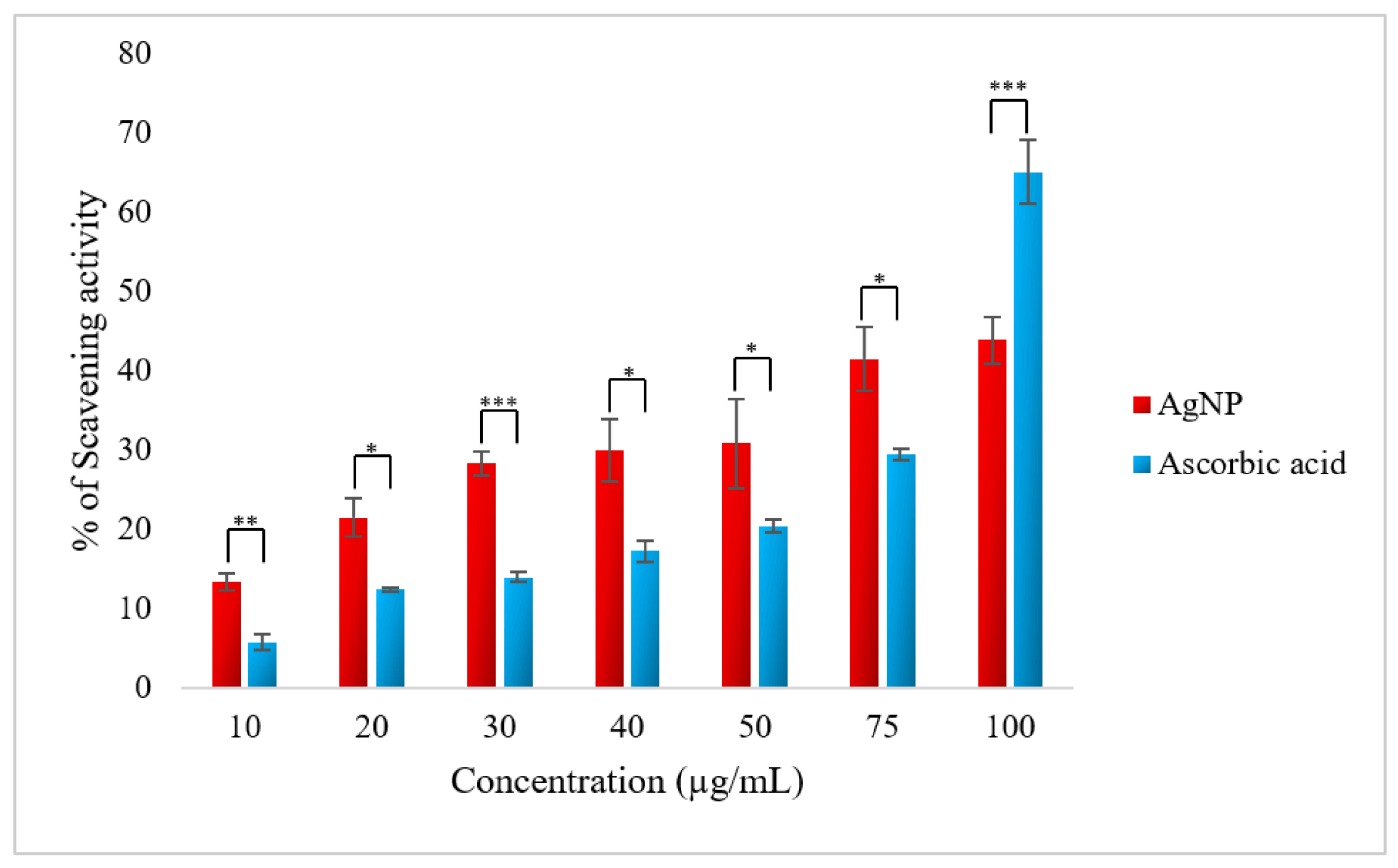
Antioxidant activity of AgNPs synthesized from *M. officinalis* flowers using the green synthesis method against DPPH free radicals. Asterisks indicate statistically significant differences between AgNPs and ascorbic acid at the corresponding concentration (*p < 0.05, ** *p* < 0.01, *** *p* < 0.001; Holm-adjusted post hoc comparisons).

DPPH is a stable free radical that can be reduced by accepting a hydrogen atom or an electron. The antioxidant potential of AgNPs obtained from *M. officinalis* flower extract using the green synthesis method was evaluated based on color change using the DPPH free radical scavenging method, and the results obtained were compared with the standard antioxidant ascorbic acid (Figure 9). The findings show that both AgNPs and ascorbic acid inhibit DPPH radicals in a concentration-dependent manner. The free radical scavenging activity of AgNPs showed a steady increase with increasing AgNP concentration. An inhibition level of 13.4 ± 1.0% was observed at a concentration of 10 µg/mL, while an inhibition level of 43.9 ± 2.9% was obtained at 100 µg/mL. Two-way ANOVA demonstrated that both sample type (AgNPs vs. ascorbic acid) and concentration had statistically significant effects on DPPH radical scavenging activity (*p* < 0.001). Furthermore, a significant interaction between sample type and concentration was observed (*p* < 0.001), indicating that the antioxidant responses of AgNPs and ascorbic acid differed depending on concentration. Post hoc pairwise comparisons with Holm correction showed statistically significant differences between AgNPs and ascorbic acid at all tested concentrations (*p* < 0.05). The inhibition percentage of AgNPs was higher than that of ascorbic acid at concentrations ranging from 10 to 75 µg/mL, whereas ascorbic acid exhibited significantly higher radical scavenging activity at 100 µg/mL. The higher radical scavenging efficiency of ascorbic acid at high concentrations can be explained by its strong electron-donating ability and fast reaction kinetics with DPPH radicals. In contrast, it has been reported that the antioxidant activity of AgNPs is largely related to phytochemical compounds (such as phenolic compounds, flavonoids, and tannins) attached to their surfaces and surface redox reactions, which may limit radical scavenging efficiency to a certain extent [51].

### 2.5. Photocatalytic Activity of the Synthesized AgNPs

**Figure 10:**
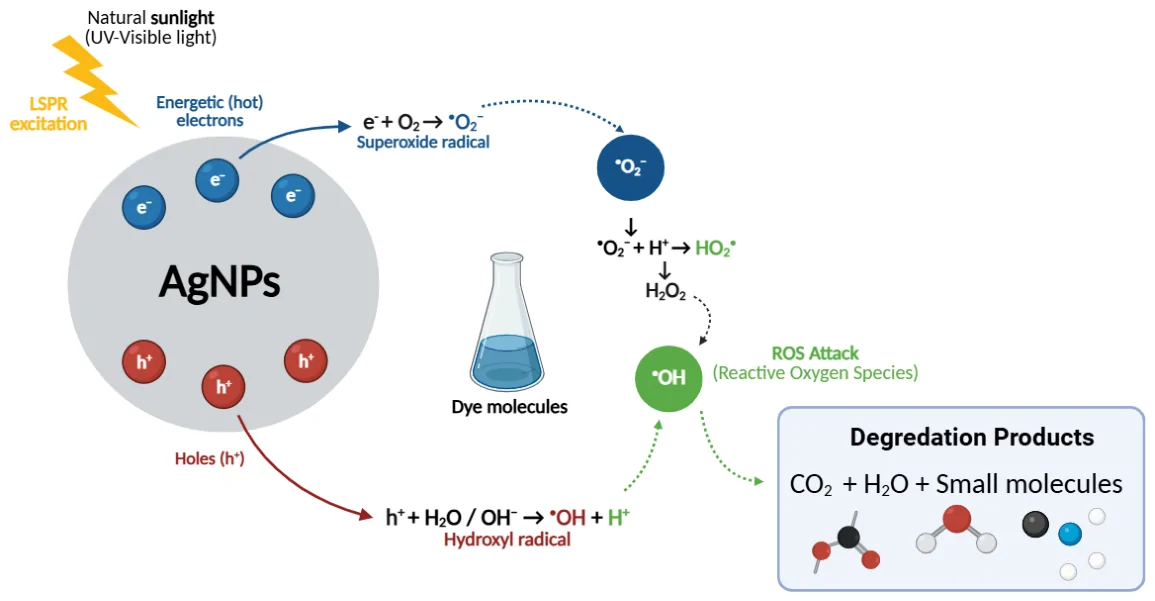
A schematic representation of the photocatalytic mechanism of AgNPs synthesized from *M. officinalis* flowers via a green synthesis method for the degradation of dyes under UV–Visible light.

It is possible to elucidate the proposed photocatalytic activity mechanism of AgNPs (Figure 10) as a light-induced redox process, in which AgNPs synthesized via a green route using *M. officinalis* flower extract facilitate dye degradation through the generation of reactive oxygen species (ROS). The first step of the photo-induced reaction is the generation of electron–hole (e^−^/h^+^) pairs. Under UV–visible light, the interaction of AgNPs with incident photons can induce localized surface plasmon resonance (LSPR) excitation, resulting in the generation of energetic (“hot”) electrons and holes that can participate in subsequent surface redox reactions [52]. In this process, the plasmonic nature of AgNPs may contribute to photocatalytic activity through LSPR, which arises from the collective oscillation of conduction electrons during interaction with incident light. LSPR can enhance the interaction of AgNPs with light, thereby supporting the formation and transfer of charge carriers at the surface and, consequently, facilitating subsequent surface redox reactions and ROS formation [52,53].

**Figure 11:**
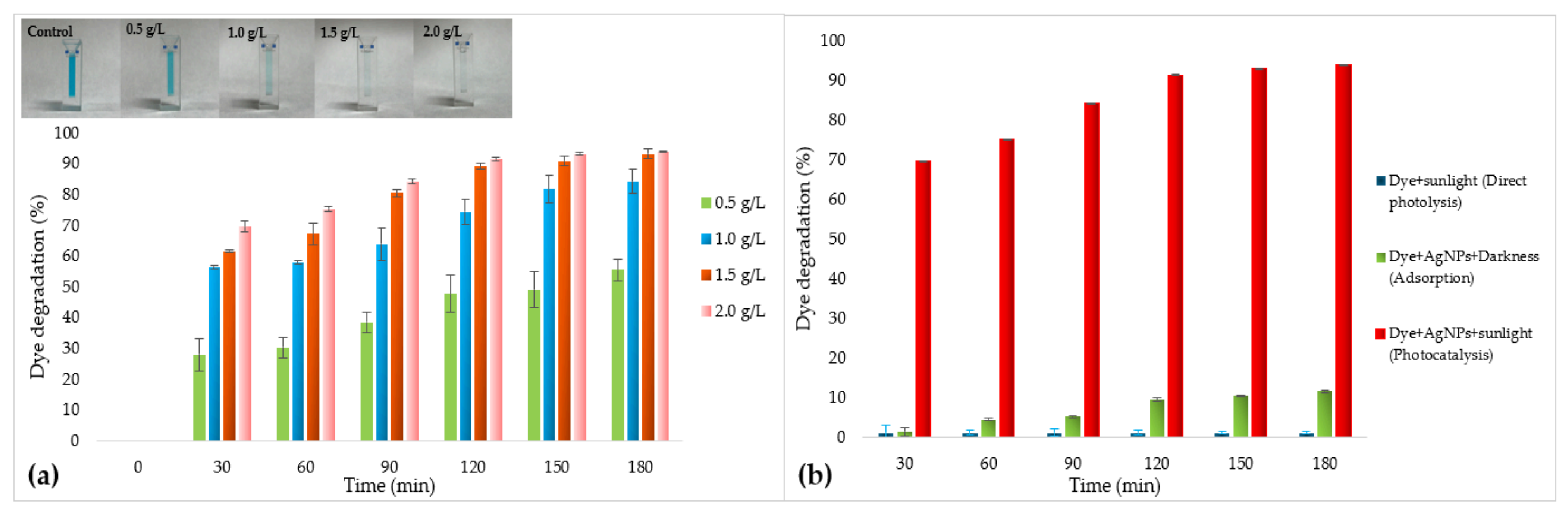
(**a**) MB dye decolorization percentages at different AgNPs concentrations at different exposure times. (**b**) Comparison of MB dye decolorization under direct photolysis, adsorption, and photocatalytic conditions using the optimized AgNP concentration (2.0 g/L).

The photogenerated energetic electrons can reduce dissolved oxygen to form superoxide radicals (•O_2_^−^), while the photogenerated holes can oxidize surface-adsorbed water or hydroxyl species, potentially leading to the formation of hydroxyl radicals (•OH) [53]. Additionally, •O_2_^−^ species can be protonated to form intermediate species such as HO_2_• and subsequently H_2_O_2_, and the decomposition of these species generates additional •OH radicals, thereby causing a chain-like increase in ROS production within the system [54]. These highly reactive ROSs may contribute to the degradation of organic dye molecules containing aromatic rings and chromophore groups by disrupting their π-conjugated structures and forming intermediate products (amines, small aromatics, etc.). However, complete mineralization into harmless end products such as CO_2_ and H_2_O cannot be confirmed based on dye decolorization measurements alone [52]. Furthermore, ROS formation and the proposed degradation pathway were not directly investigated in the present study. The phytochemical coating on the surface of AgNPs synthesized using plant extracts not only facilitates the approach of dye molecules to the catalyst surface by providing adsorption sites but also stabilizes charge separation, thereby reducing electron–hole recombination and thus enhancing photocatalytic efficiency [52]. The results presented in Figure 11a show that the effectiveness of AgNPs obtained from *M. officinalis* flowers via the green synthesis method in the photocatalytic degradation of MB dye increases significantly depending on both NP concentration and contact time. It is observed that the dye removal percentage increases steadily over time at all concentrations, with a particularly rapid increase occurring in the first 30 min; this phenomenon can likely be attributed to high initial surface adsorption and active radical formation. At the lowest concentration of 0.5 g/L, the maximum degradation remained at 55.4 ± 3.4%, while increasing the concentration to 1.0 and 1.5 g/L resulted in values of 84.1 ± 3.8% and 93.1 ± 1.5%, respectively. The highest efficiency was achieved at an AgNP concentration of 2.0 g/L, resulting in 93.5 ± 0.3% color removal after 180 min. To distinguish photocatalytic decolorization from direct photolysis and adsorption, control experiments were performed using the optimized AgNP concentration of 2.0 g/L (Figure 11b). The direct photolysis control consisted of the dye solution exposed to natural sunlight in the absence of AgNPs, while the adsorption control consisted of the dye solution containing AgNPs maintained under dark conditions. Two-way ANOVA revealed that both irradiation time (*F*_6,28_ = 749.96, *p* < 0.001) and AgNP concentration (*F*_3,28_ = 458.89, *p* < 0.001) significantly affected the photocatalytic degradation efficiency of MB. Moreover, the interaction between irradiation time and AgNP concentration was statistically significant (*F*_18,28_ = 13.88, *p* < 0.001), indicating that the influence of catalyst concentration on MB degradation varied with reaction time. These results statistically confirm that enhanced photocatalytic performance resulted from the combined effects of increasing catalyst dosage and prolonged irradiation. This indicates that an increased NP concentration provides a larger active surface area and facilitates the production of more ROS for photocatalytic reaction. However, the slowing of the increase rate beyond a certain point at high concentrations suggests that limiting effects, such as light scattering or particle agglomeration, may arise in the system. Overall, the findings indicate that green-synthesized AgNPs have significant potential as an effective and eco-friendly photocatalyst for the removal of MB dye under natural sunlight.

**Figure 12:**
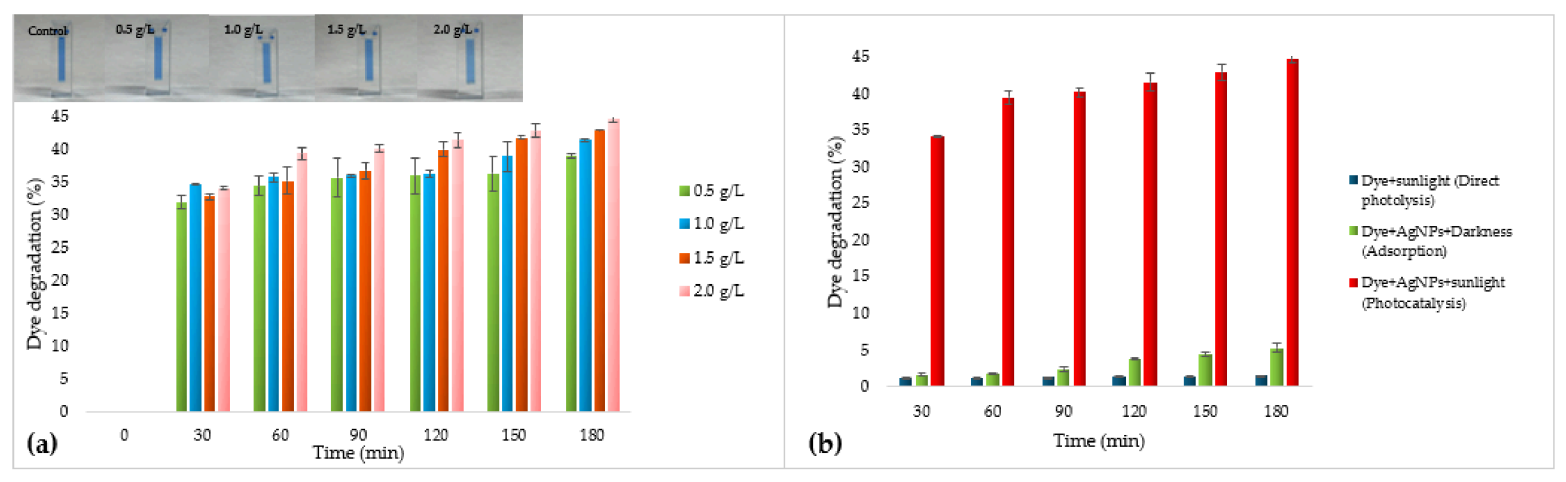
(**a**) CBB R-250 dye decolorization percentages at different AgNPs concentrations at different exposure times. (**b**) Comparison of CBB R-250 dye decolorization under direct photolysis, adsorption, and photocatalytic conditions using the optimized AgNP concentration (2.0 g/L).

The results presented in Figure 12a clearly demonstrate that AgNPs obtained from *M. officinalis* flowers via a green synthesis method are effective in the photocatalytic degradation of CBB R-250 dye. Upon examination of the data, rapid color removal occurred within the first 30 min at all concentrations; this is related to the rapid adsorption of dye molecules onto nanoparticles with high surface area, followed by the initiation of photocatalytic reactions. A gradual increase in the color removal percentage was observed as the contact time increased; in particular, the highest degradation efficiency of 44.8 ± 0.6% was achieved at a 2.0 g/L AgNP concentration after 180 min. To distinguish photocatalytic decolorization from direct photolysis and adsorption, control experiments were performed using the optimized AgNP concentration of 2.0 g/L (Figure 12b). The direct photolysis control showed negligible CBB R-250 decolorization throughout the experimental period, whereas the adsorption control resulted in a limited increase in dye removal, reaching 5 ± 0.6% after 180 min. Two-way ANOVA demonstrated that irradiation time (*F*_6,28_ = 1073.39, *p* < 0.001) and AgNP concentration (*F*_3,28_ = 27.29, *p* < 0.001) had significant effects on the photocatalytic degradation efficiency of CBB R-250. In addition, a significant interaction between irradiation time and AgNP concentration was observed (*F*_18,28_ = 2.32, *p* = 0.0219), indicating that the effect of AgNP concentration differed significantly across irradiation times. These statistical results confirm that the observed improvement in photocatalytic performance was governed by the combined effects of catalyst dosage and reaction time rather than random experimental variation. The increase in photocatalytic activity parallel to the rise in concentration can be explained by the provision of a larger active surface area and more reaction sites. However, the fact that the increase remains limited beyond a certain point can be attributed to factors such as light scattering and particle agglomeration. Overall, the results demonstrate that AgNPs can be used as an effective photocatalyst under natural sunlight and that both contact time and nanoparticle concentration are critical parameters in dye removal.

It is thought that the observed photocatalytic behavior may have been significantly influenced by interactions between the charge properties of the dye molecules and the surface properties of the AgNPs synthesized using *M. officinalis* extract. DLS and zeta potential analyses have revealed that the synthesized AgNPs are negatively charged nanoparticles with a zeta potential of −73.2 mV, exhibiting high colloidal stability. Whilst this negative surface charge may have facilitated the more effective adsorption of cationic MB molecules onto the AgNP surface via electrostatic attraction, it may have limited the interaction of anionic CBB R-250 molecules with the catalyst surface due to electrostatic repulsion [55,56,57]. It is considered that this difference in surface adsorption is one of the key factors determining the degradation efficiency, as it directly affects the contact between the ROS and the dye molecules formed during the photocatalytic reaction [58,59].

The findings indicate that AgNPs synthesized via a green synthesis method using *M. officinalis* flower extract can be considered an effective photocatalyst for the photocatalytic degradation of organic dyes. However, photocatalytic performance is a multiparameter process shaped by the combined effects of the nanoparticles’ physicochemical properties, the molecular structure of the target dye, and the experimental conditions [60]. Therefore, to enable a more comprehensive evaluation of the results obtained, selected literature studies examining the photocatalytic activity of AgNPs synthesized via green synthesis using plant extracts against various organic dyes are presented in a comparative manner in Table 2.

### 2.6. Antimicrobial Activity of the Synthesized AgNPs

**Figure 13:**
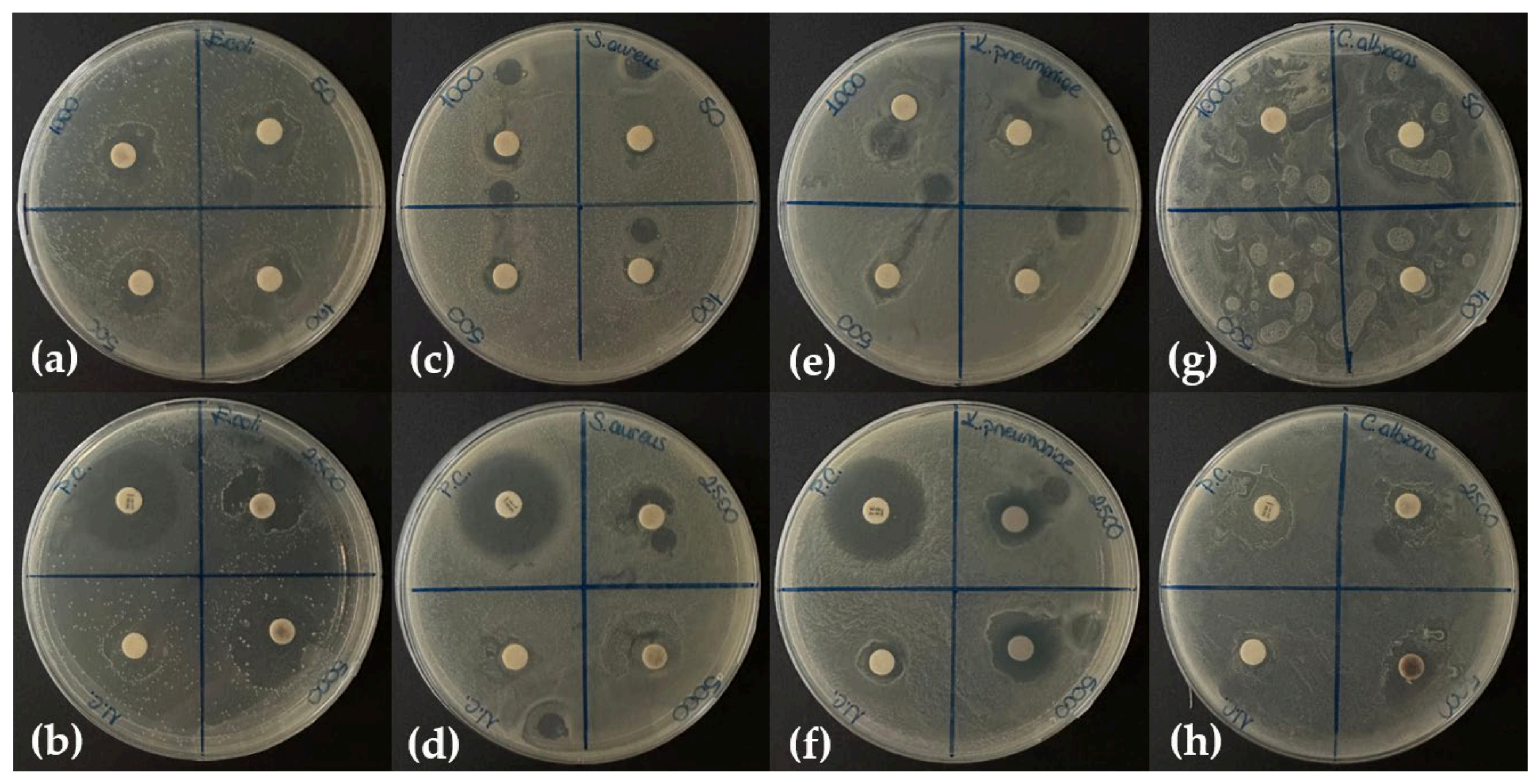
Antimicrobial activity of AgNPs synthesized from *M. officinalis* flowers using the green synthesis method. [(**a**,**b**) *E. coli*; (**c**,**d**) *S. aureus*; (**e**,**f**) *K. pneumoniae*; (**g**,**h**) *C. albicans*; with concentrations of 50, 100, 500, 1000, 2500, and 5000 µg/mL] (PC: positive control as tetracycline (10 µg); NC: negative control as DMSO).

The antimicrobial activity of AgNPs synthesized from *M. officinalis* flowers via a green synthesis method was evaluated by the disk diffusion method. The results showed that the NPs exhibited concentration-dependent inhibition against all tested microorganisms (Figure 13) (Table 3). At a concentration of 5000 µg/mL, zones with diameters of 2.20 cm and 2.00 cm were obtained against *E. coli* and *S. aureus*, respectively. Inhibition of *K. pneumoniae* was observed at lower concentrations, with a zone diameter of 1.90 cm at 5000 µg/mL. Antimicrobial effects on *C. albicans* were seen only at high concentrations of 2500 and 5000 µg/mL. However, these findings should be interpreted with caution, particularly for *C. albicans*, for which inhibition was observed only at relatively high AgNPs concentrations. Moreover, the disk diffusion assay provides a preliminary assessment of antimicrobial activity and does not allow direct determination of the minimum inhibitory concentration (MIC), minimum bactericidal concentration (MBC), or minimum fungicidal concentration (MFC). Therefore, further studies using MIC, MBC, and MFC assays are warranted to provide a more comprehensive evaluation of the antimicrobial efficacy of the synthesized AgNPs. The concentration-dependent antimicrobial activity observed in the study can be explained by the multifaceted mechanism of action of AgNPs [70].

AgNPs adsorb to the surface of microbial cells, disrupting the structural integrity of the cell wall and cytoplasmic membrane, and increasing membrane permeability, thereby causing the cell contents to leak into the external environment [71]. In addition, Ag^+^ ions released from nanoparticles enter the cell, interact with sulfhydryl (-SH) groups in proteins, inhibit enzymatic activity, and suppress cellular metabolism [72]. Furthermore, by increasing ROS production, they cause oxidative damage to lipids, proteins, and DNA, ultimately leading to the loss of cellular integrity and triggering cell death [70,73]. The fact that AgNPs are effective against multiple cellular targets simultaneously is considered one of the main reasons they exhibit broad-spectrum antimicrobial activity against Gram-positive and Gram-negative bacteria as well as fungal species [73]. In comparison to previous studies on green-synthesized AgNPs from various plant extracts, including even the *M. officinalis* (Table 4), AgNPs synthesized from *M. officinalis* flowers (in this study) exhibited concentration-dependent antimicrobial activity against Gram-positive (*S. aureus*) and Gram-negative (*E. coli* and *K. pneumoniae*) bacterial and fungal (*C. albicans*) species. Notably, these values are higher than those reported, and these findings demonstrate the concentration-dependent antimicrobial activity of the synthesized AgNPs under the experimental conditions employed.

## 3. Materials and Methods

All chemicals used in this study were high-quality analytical grade and used without further purification. Silver nitrate (AgNO_3_, 99.9% Merck) was used in this study. The Gram-negative microorganisms *Escherichia coli* (ATCC 8739) and *Klebsiella pneumoniae* (DSM 789), along with the Gram-positive bacterium *Staphylococcus aureus* (DSM 799), were acquired from the Deutsche Sammlung von Mikroorganismen und Zellkulturen, DSMZ, Germany. The fungus *Candida albicans* (ATCC 10231) was acquired from the American Type Culture Collection located in Manassas, VA, USA.

### 3.1. Preparation of Plant Extract

Fresh flowers of *M. officinalis* were collected from Kemalpaşa, İzmir, Türkiye (38°25′57.4″ N, 27°26′08.7″ E). The plant material was formally identified by Dr. Emre Erden Kopar based on morphological characteristics. The taxonomic nomenclature was verified using the GBIF and Catalogue of Life databases. No voucher specimen number is currently available.

The plant material was thoroughly washed with distilled water, cleaned, and dried at room temperature. Dried samples were mechanically ground into powder. Five grams of powdered plant material were extracted with 50 mL of 80% methanol at 60 °C for 2 h. After cooling to room temperature, the extract was filtered through Whatman filter paper and stored at 4 °C until further analysis.

#### Identification and Quantification of Phytochemical Compounds by HPLC-DAD

The identification and quantification of phytochemical compounds in an 80% methanol extract of *M. officinalis* flowers were performed using an Agilent Technologies 1260 Infinity high-performance liquid chromatography (HPLC-DAD) system (Agilent Technologies, Santa Clara, CA, USA) equipped with a diode array detector (DAD). Chromatographic separation was performed using a reverse-phase C18 column (ZORBAX Eclipse Plus C18, Agilent Technologies, Santa Clara, CA, USA). The mobile phase consisted of water (A) containing 0.1% phosphoric acid and acetonitrile (C). The gradient elution program was as follows: 83–85% A from 0 to 7 min, 85–80% A from 7 to 20 min, 20–24 min: 80–75% A; 24–28 min: 75–70% A; 28–30 min: 70–60% A; 30–32 min: 60–50% A; 32–36 min: 50–30% A; and 36–40 min: 30–83% A. The flow rate was set to 0.8 mL/min, the column temperature to 30 °C, and the injection volume to 10 µL. The analyses were performed at wavelengths of 200 and 300 nm, using reference wavelengths of 500 and 100 nm. Compounds were identified by comparing the retention times of the reference standards with the peaks in the sample chromatogram; quantification was performed using calibration curves based on external standards. Results were expressed in ng/µL.

### 3.2. Synthesis of Silver Nanoparticle (AgNPs)

**Figure 14:**
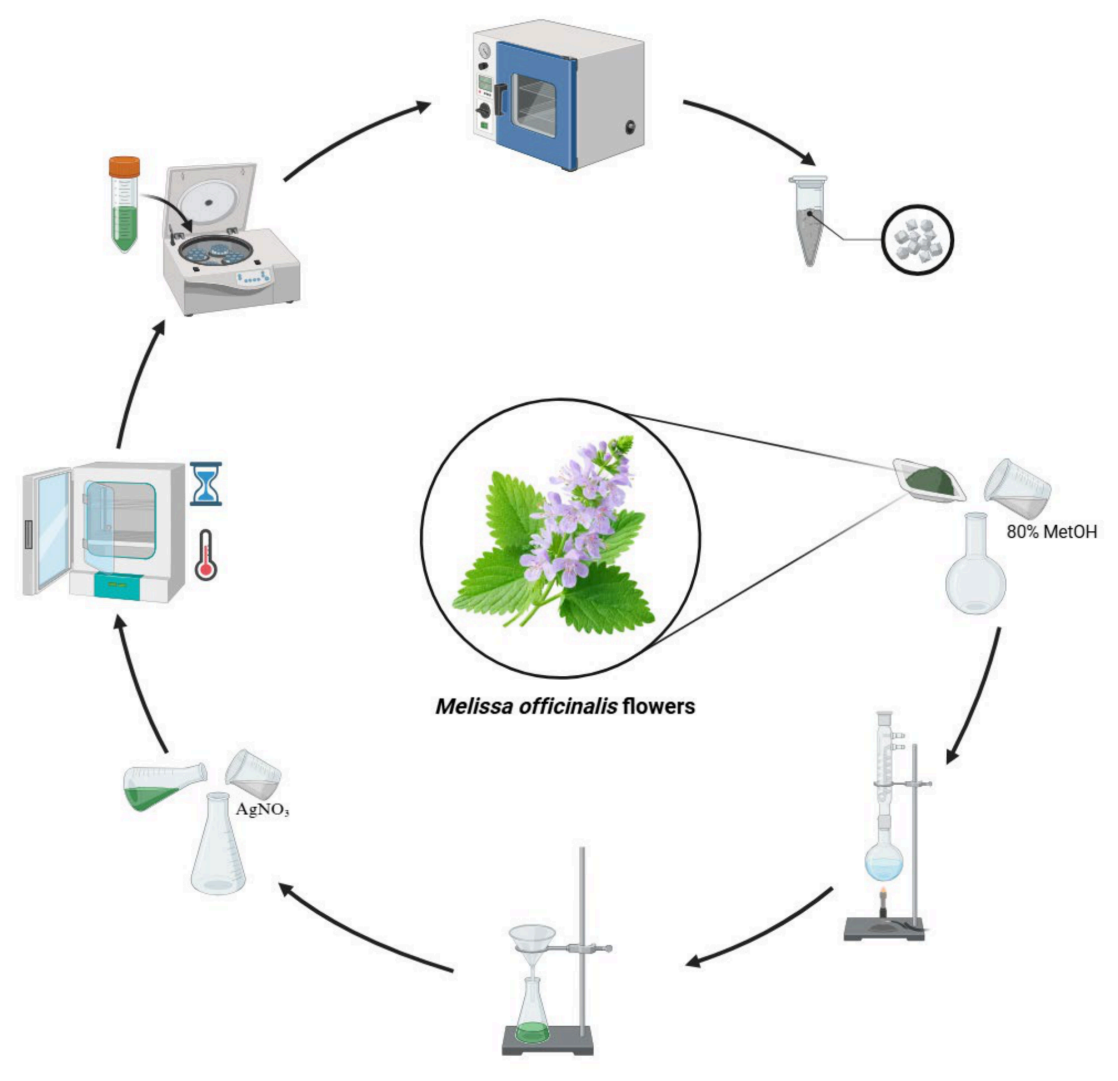
A schematic diagram illustrating the synthesis of AgNPs from *M. officinalis* flowers using the green synthesis method.

AgNPs were synthesized by reducing AgNO_3_ solution with phenolic compounds from the methanol extract of *M. officinalis* flowers [80]. The reaction mixture was prepared by adding 50 mL of AgNO_3_ solution and 5 mL of plant extract to a 100 mL erlenmeyer flask. In the synthesis process, key parameters such as temperature (30, 45, and 60 °C), initial pH values (5, 7, and 9), reaction time (1, 3, 9, 12, and 24 h), and AgNO_3_ concentration (1, 5, and 10 mM) were tested at various levels to determine their effects on nanoparticle formation. During the evaluation of each parameter, the synthesis conditions other than the parameter under investigation were kept constant. The optimal conditions were determined based on the UV-Vis spectral properties of the synthesized AgNPs, particularly the intensity and position of the characteristic SPR band. The reactions were carried out under continuous stirring, and nanoparticle formation was monitored by color change. After the synthesis was completed, the resulting AgNPs were centrifuged at 10,000 rpm for 10 min; they were then purified by washing several times with distilled water and ethanol to remove impurities. The obtained pure nanoparticles were dried at 60 °C for further characterization steps (Figure 14).

### 3.3. Characterization of Silver Nanoparticles (AgNPs)

#### 3.3.1. UV-Visible Spectroscopy Analysis

A preliminary study of AgNPs in colloidal solution was developed and analyzed using Lambda 25 UV-visible spectrophotometer (Perkin Elmer, Shelton, CT, USA), and spectral scanning was performed in the wavelength range of 200–800 nm.

#### 3.3.2. ATR-FTIR Analysis

To identify the functional groups involved in the AgNP synthesis and stabilization processes, Fourier transform infrared (ATR-FTIR) spectroscopy was performed on both plant and NP samples. Measurements were performed in attenuated total reflection (ATR) mode using an IRSpirit™ FTIR spectrometer (Shimadzu, Kyoto, Japan) equipped with a diamond ATR crystal. Spectra were collected in the range of 4000–400 cm^−1^ at a resolution of 4 cm^−1^. This method allowed for the identification of specific chemical groups associated with nanoparticle formation and stabilization.

#### 3.3.3. Scanning Electron Microscope Analysis (SEM)

The morphology, shape properties, and surface structure of AgNPs have been studied in detail using the Apreo S field emission scanning electron microscope (SEM) (Thermo Fisher Scientific, Waltham, MA, USA). Prior to SEM analysis, the dried AgNP samples were placed on a sample holder and coated with a thin layer of gold to enhance electrical conductivity during imaging and minimize the effects of charge accumulation.

#### 3.3.4. Dynamic Light Scattering (DLS) and Zeta Potential Analysis

The hydrodynamic particle size distribution and surface charge (zeta potential) of the produced AgNPs were assessed using a HORIBA Nanopartica SZ-100V2 instrument (HORIBA Ltd., Kyoto, Japan). For sample preparation, the obtained AgNPs were dried at 60 °C and subsequently dispersed in 20 mM HEPES buffer (pH 7.4) at a concentration of 10 mg/mL. The samples were homogenized by vortex mixing and centrifuged, followed by dilution at a ratio of 1:100 with HEPES buffer. The diluted suspension was transferred into a polystyrene cuvette for hydrodynamic particle size measurement. For zeta potential analysis, 120 µL of the diluted suspension was transferred into a zeta potential cell. Measurements were conducted at ambient temperature. The hydrodynamic particle size was determined using dynamic light scattering (DLS) analysis, whilst the surface charge and colloidal stability of the nanoparticles were examined via zeta potential analysis.

#### 3.3.5. X-Ray Diffraction Analysis (XRD)

The crystalline structure, phase composition, and crystallinity of the AgNPs were analyzed using an X-ray diffractometer (D-Max 2200 PC, Rigaku Corporation, Tokyo, Japan) operating with Cu–Kα radiation (*λ* = 0.15406 nm). Diffraction patterns were recorded over a 2θ range of 3–90°. The average crystallite size of the AgNPs was estimated using the Debye–Scherrer equation (Equation (1)):

(1) $$D=\frac{K\cdot \lambda \;}{\beta \cdot cos\theta }$$

where *D* is the average crystallite size (nm), *K* is the shape factor (0.9), *λ* is the wavelength of the Cu–Kα radiation (0.15406 nm), *β* is the full width at half maximum (FWHM) of the diffraction peak expressed in radians, and *θ* is the Bragg diffraction angle. The crystallite size was calculated from the characteristic diffraction peaks of the AgNPs using their corresponding FWHM values [80].

### 3.4. Determination of Antioxidant Activity with 2,2-Diphenyl-1-Picrylhydrazyl (DPPH) Radical Scavenging Assay

The 1,1-Diphenyl-2-picrylhydrazyl (DPPH) free radical scavenging activity of AgNPs obtained by green synthesis using *M. officinalis* flower extract was determined according to the method reported by Keshari et al. [25]. AgNP solutions prepared at different concentrations (10, 20, 30, 40, 50, 75, and 100 µg/mL) and ascorbic acid as a standard antioxidant were placed in separate test tubes. One milliliter of each sample was taken, and 1 mL of DPPH solution (0.1 mM) prepared in methanol was added to it, and the mixture was homogenized by vortexing. The mixtures were incubated at room temperature in the dark for 30 min. After incubation, the absorbance of the stable DPPH radical was measured at 517 nm. A DPPH solution without a sample was prepared as a control following the same procedure. A sample blank containing the corresponding AgNP solution and methanol without DPPH was also prepared to account for the possible optical interference of AgNPs at 517 nm. The blank-corrected absorbance values were used for the calculation of DPPH radical scavenging activity. The free radical scavenging activity was expressed as the inhibition percentage (%) calculated using the following equation [81]:

$$DPPH\;radical\;scavenging\;activity\;(\%)=\left(\;\frac{A_{c}-A_{s}}{A_{c}}\right)\;\times \;100,$$

where *A_c_* is the control absorbance of DPPH radical and methanol, and *A_s_* is the sample absorbance of DPPH radical and sample AgNPs/standard ascorbic acid.

### 3.5. Photocatalytic Activity

To evaluate the photocatalytic activity of AgNPs synthesized from the flowers of *M. officinalis* using the green synthesis method, the degradation of cationic CBB R-250 and anionic MB dyes was examined at different AgNP concentrations (0.50, 1.0, 1.5, and 2.0 g/L) and different contact times (30 min, 1 h, 1.5 h, 2 h, 2.5 h, and 3 h) under natural sunlight. The photocatalytic experiments were performed in triplicate using 10 mL of dye solution at a concentration of 10 ppm, mixed with different AgNP concentrations, and incubated under shaking conditions at an ambient temperature of approximately 26 °C. The experiments were conducted under natural sunlight between 12:00 p.m. and 3:00 p.m. Before exposure to sunlight, the reaction mixtures were stirred in the dark for 30 min to establish adsorption/desorption equilibrium. This dark equilibration step was used to minimize the contribution of initial adsorption to the subsequent sunlight-induced dye removal. Control experiments were performed to distinguish photocatalytic degradation from direct photolysis and adsorption. The dye solution exposed to natural sunlight in the absence of AgNPs was used as the direct photolysis control, while the dye solution containing AgNPs and maintained under dark conditions was used as the adsorption control. The dye solution containing AgNPs and exposed to natural sunlight was considered the photocatalytic system. After each photocatalytic run, the nanoparticles were separated from the suspension by centrifugation at 5000 rpm for 5 min. Subsequently, the optical density (OD) of the dye solutions (CBB R-250 at 590 nm and MB at 664 nm) was measured using a UV–Vis spectrophotometer (Perkin Elmer), and the color removal efficiency (%) was calculated using the following equation [82]:

$$D\;\left(\%\right)=(\frac{{Dye}_{\left(i\right)}-{Dye}_{\left(f\right)}}{{Dye}_{\left(i\right)}})\;\times \;100$$

where *Dye*_(*i*)_ and *Dye*_(*f*)_ represent the initial and final absorbance values, respectively.

### 3.6. Antimicrobial Activity

The disk diffusion method was used to investigate the antimicrobial activity of the synthesized AgNPs. Broth medium was used to subculture microorganisms, which were incubated at 28 °C for 24 h, and then the cultures were harvested overnight. The suspension of each test strain was standardized according to the 0.5 McFarland standard (1.5 × 10^8^ CFU/mL) and then spread onto the surface of sterile Nutrient Agar (NA) plates using sterile beads to obtain a homogeneous microbial growth plate. The bacterial strains were Gram-negative *E. coli* and *K. pneumoniae*, Gram-positive *S. aureus*, and the fungus was *C. albicans*. For the antimicrobial activity study, dimethyl sulfoxide (DMSO) was selected as the negative control and the antibiotic [tetracycline (10 µg)] as the positive control group. Tetracycline (10 µg) was used as the positive control for the bacterial strains, whereas an appropriate antifungal positive control was not included for *C. albicans*. The absence of an appropriate antifungal positive control is acknowledged as a limitation of the antimicrobial activity assessment. Finally, the Petri dishes were incubated at 37 °C for 24 h. To evaluate the antimicrobial activity of the synthesized AgNPs, the diameter of the inhibition zone was measured and compared with that of the control groups.

### 3.7. Statistical Analysis

All experiments were conducted in three independent replicates (*n* = 3), and results are presented as mean ± standard deviation (SD). Statistical analyses were performed using GraphPad Prism 10.0 software, and differences between groups were assessed using two-way analysis of variance (Two-way ANOVA). A *p*-value of <0.05 was considered statistically significant.

## 4. Conclusions

In this study, AgNPs were successfully synthesized using an eco-friendly and sustainable approach involving *M. officinalis* flower extract, and the optimal synthesis conditions were determined. The characterization analyses conducted demonstrated that AgNPs stabilized with plant-derived biomolecules, possessing a crystalline structure and exhibiting high colloidal stability, were successfully obtained. The synthesized AgNPs exhibited concentration-dependent antioxidant and antimicrobial activities against Gram-positive and Gram-negative bacteria as well as fungal microorganisms. Furthermore, it was determined that MB was photocatalyzed at a high rate under natural sunlight, while the CBB R-250 dye was photocatalyzed at a lower rate. Statistical analyses have confirmed the significant effect of nanoparticle concentration and irradiation time on photocatalytic performance, and the significant effect of nanoparticle concentration on antioxidant activity.

When considering the existing studies in the literature, this study is one of a limited number of comprehensive studies that simultaneously evaluate the synthesis optimization, detailed physicochemical characterization, and antioxidant, antimicrobial, and photocatalytic properties of AgNPs synthesized from *M. officinalis* flowers. The findings indicate that *M. officinalis* flowers can serve as a biological source for the synthesis of AgNPs and that the synthesized AgNPs exhibit antioxidant, antimicrobial, and photocatalytic activities under the experimental conditions investigated. Given the plant-extract-mediated synthesis approach and the observed antioxidant, antimicrobial, and photocatalytic activities, the synthesized AgNPs may be considered as candidates for further investigation in biomedical and environmental applications, particularly in antimicrobial and photocatalytic systems.

## Figures and Tables

**Table 1:** Crystallite sizes of AgNPs via green synthesis *M. officinalis* flower extract, calculated using the Debye–Scherrer equation.

2θ (°)	hkl Plane	FWHM (°)	β (rad)	Crystallite Size (nm)
38.0	(111)	0.804	0.01403	10.45
44.2	(200)	1.057	0.01845	8.10
64.6	(220)	1.139	0.01988	8.25
77.5	(311)	1.075	0.01876	9.46
Average ± SD				9.07 ± 1.11

Reference: JCPDS No. 04-0783 for metallic Ag (fcc structure).

**Table 2:** Comparison of the photocatalytic degradation performance of green-synthesized AgNPs against various dyes reported in the literature and in the present study.

Plant Extract	Dye	Concentration of Catalyst	Concentration of Dye	Degradation Time	Dye Degradation (%)	References
*Delonix elata*	MB	20 mg	20 mg/mL	120 min	85%	[61]
*Ficus elastica*	CR	10 µL	10 mg/mL	10 min	91.21%	[62]
*Citrus reticulata Blanco*	MG	20 mg	100 mg/mL	5 days	100%	[63]
*Callistemon lanceolatus*	MB	0.1 mg/mL	50 mg/L	300 min	91%	[64]
*Terminalia arjuna*	MO	1 mM	10 µL	14 min	86.68%	[65]
	MB	1 mM	20 µL	19 min	93.60%	
	CR	1 mM	20 µL	14 min	92.20%	
	4-NP	5 mM	20 µL	15 min	88.80%	
*Eucalyptus globulus*	MO	3 mg/L	50 mg/mL	10 min	>90%	[66]
	MR			20 min		
	CR			20 min		
*Plantago ovata*	MB	0.05%	10 mM	18 min	100%	[67]
	CR			20 min		
*Eulophia herbacea (Lindl.)*	MB	7 µg/mL	10 mM	30 min	~100%	[68]
	CR		1 mM			
*Alocasia macrorrhiza*	4-NP	0.2 mg/mL	0.076 mM	70 s	100%	[69]
	2,4-DNPH	0.2 mg/mL	2.5 mM	1 s		
	MO	2.5 mg/mL	3.0 × 10^−5^ M	2 s		
	CR	3.5 mg/mL	1.44 × 10^−5^ M	47 s		
	NS	2.5 mg/mL	1.62 × 10^−4^ M	45 s		
*M officinalis flowers*	MB	2 g/L	10 ppm	180 min	93.5 ± 0.3%	This work
	CBB R-250				44.8 ± 0.6%	

**Table 3:** Inhibition zones of AgNPs synthesized from *M. officinalis* flowers using the green synthesis method, as demonstrated by the disk diffusion method on various microorganisms.

Pathogenic Microorganism	Zone of Inhibition (mm)						
	P.C. (Tetracycline (10 µg))	50 µg/mL	100 µg/mL	500 µg/mL	1000 µg/mL	2500 µg/mL	5000 µg/mL
*E. coli*	29.0	16.0	16.5	17.0	17.5	20.0	22.0
*S. aureus*	25.5	14.5	15.5	17.0	18.0	19.0	20.0
*K. pneumoniae*	25.0	10.5	12.5	13.5	14.0	17.0	19.0
*C. albicans*	-	-	-	-	-	14.5	17.0

**Table 4:** Antimicrobial activity of AgNPs synthesized from various plant extracts.

Plant Extract	Microbial Strains	Antimicrobial Activity (Inhibition Zone (mm))	References
*Melissa officinalis*	*B. subtilis*	5.7	[21]
	*S. aureus*	5.6	
	*E. coli*	7	
	*S. cerevisiae*	4	
*Melissa officinalis*	*S. aureus*	11.5	[19]
	*E. coli*	12.5	
*Calendula officinalis*	*E. coli*	10.50 ± 0.35	[74]
	*S. aureus*	15.10 ± 0.10	
	*K. pneumoniae*	10.0 ± 0.10	
*Pu-erh tea* leaves	*E. coli*	15	[75]
	*K. pneumoniae*	10	
	*S. typhimurium*	20	
	*S. enteritidis*	20	
*Eucalyptus viminalis Labill*	*E. coli*	0	[76]
	*E. faecalis*	0	
	*S. aureus*	8.5 ± 2.5	
	*P. aeruginosa*	10.5 ± 0.5	
*Carya illinoinensis*	*S. aureus*	11	[77]
	*L. monocytogenes*	12	
	*E. coli*	15	
	*P. aeruginosa*	13	
*Vigna mungo*	*E. coli*	19 ± 0.95	[78]
	*S. aureus*	20 ± 0.95	
*Althaea officinalis* flowers	*E. coli*	19	[79]
	*S. aureus*	22	
	*K. pneumoniae*	18	
	*C. albicans*	16.5	

## Data Availability

All results generated or analyzed during this study are fully presented in the manuscript.
